# *Akkermansia muciniphila* and *Parabacteroides distasonis* synergistically protect from colitis by promoting ILC3 in the gut

**DOI:** 10.1128/mbio.00078-24

**Published:** 2024-03-12

**Authors:** Joana Gaifem, Ana Mendes-Frias, Mathis Wolter, Alex Steimle, Maria Jose Garzón, Carles Ubeda, Clarisse Nobre, Abigail González, Salomé S. Pinho, Cristina Cunha, Agostinho Carvalho, António Gil Castro, Mahesh S. Desai, Fernando Rodrigues, Ricardo Silvestre

**Affiliations:** 1 Life and Health Sciences Research Institute (ICVS), School of Medicine, University of Minho, Braga, Portugal; 2 ICVS/3B’s – PT Government Associate Laboratory, Braga/Guimarães, Portugal; 3 i3S – Institute for Research and Innovation in Health, University of Porto, Porto, Portugal; 4 Department of Infection and Immunity, Luxembourg Institute of Health, Esch-sur-Alzette, Luxembourg; 5 Faculty of Science, Technology and Medicine, University of Luxembourg, Esch-sur-Alzette, Luxembourg; 6 Fundación para el Fomento de la Investigación Sanitaria y Biomédica de la Comunitat Valenciana (FISABIO), Valencia, Spain; 7 Centers of Biomedical Research Network (CIBER) in Epidemiology and Public Health, Madrid, Spain; 8 Centre of Biological Engineering (CEB), University of Minho, Campus de Gualtar, Braga, Portugal; 9 LABBELS – Associate Laboratory, Braga/Guimarães, Portugal; 10 ICBAS-School of Medicine and Biomedical Sciences, University of Porto, Porto, Portugal; 11 Faculty of Medicine, University of Porto, Porto, Portugal; 12 Odense Research Center for Anaphylaxis, Department of Dermatology and Allergy Center, Odense University Hospital, University of Southern Denmark, Odense, Denmark; University of Chicago, Chicago, Illinois, USA

**Keywords:** *Akkermansia muciniphila*, *Parabacteroides distasonis*, colitis, microbiome, ILC, gut immunity

## Abstract

Inflammatory bowel disease (IBD) is a group of inflammatory conditions of the gastrointestinal tract. The etiology of IBD remains elusive, but the disease is suggested to arise from the interaction of environmental and genetic factors that trigger inadequate immune responses and inflammation in the intestine. The gut microbiome majorly contributes to disease as an environmental variable, and although some causative bacteria are identified, little is known about which specific members of the microbiome aid in the intestinal epithelial barrier function to protect from disease. While chemically inducing colitis in mice from two distinct animal facilities, we serendipitously found that mice in one facility showed remarkable resistance to disease development, which was associated with increased markers of epithelial barrier integrity. Importantly, we show that *Akkermansia muciniphila* and *Parabacteroides distasonis* were significantly increased in the microbiota of resistant mice. To causally connect these microbes to protection against disease, we colonized susceptible mice with the two bacterial species. Our results demonstrate that *A. muciniphila* and *P*. *distasonis* synergistically drive a protective effect in both acute and chronic models of colitis by boosting the frequency of type 3 innate lymphoid cells in the colon and by improving gut epithelial integrity. Altogether, our work reveals a combined effort of commensal microbes in offering protection against severe intestinal inflammation by shaping gut immunity and by enhancing intestinal epithelial barrier stability. Our study highlights the beneficial role of gut bacteria in dictating intestinal homeostasis, which is an important step toward employing microbiome-driven therapeutic approaches for IBD clinical management.

**IMPORTANCE:**

The contribution of the gut microbiome to the balance between homeostasis and inflammation is widely known. Nevertheless, the etiology of inflammatory bowel disease, which is known to be influenced by genetics, immune response, and environmental cues, remains unclear. Unlocking novel players involved in the dictation of a protective gut, namely, in the microbiota component, is therefore crucial to develop novel strategies to tackle IBD. Herein, we revealed a synergistic interaction between two commensal bacterial strains, *Akkermansia muciniphila* and *Parabacteroides distasonis*, which induce protection against both acute and chronic models of colitis induction, by enhancing epithelial barrier integrity and promoting group 3 innate lymphoid cells in the colonic mucosa. This study provides a novel insight on how commensal bacteria can beneficially act to promote intestinal homeostasis, which may open new avenues toward the use of microbiome-derived strategies to tackle IBD.

## INTRODUCTION

The gastrointestinal tract harbors a vast community of microbes, from bacteria to fungi and viruses, that have co-evolved and developed mutualistic interactions with the host. These microorganisms play a role in the complex environment that is found in the intestine, such as the occupation of niches (thus avoiding pathogen colonization) and by the synthesis of vitamins, metabolites, and other nutrients that are only accessible to the host through microbial metabolism (1). Besides acting as a defense mechanisms against infections, several pathogen recognition receptors sense and respond to the microbiota in a beneficial relationship with the host toward homeostasis and thus mediate the interaction with the intestinal epithelial barrier (1). In turn, the presence of the intestinal epithelial barrier prevents excessive contact between microorganisms and the immune cells (2). In this sense, the interaction between the microbiota, the intestinal barrier function, and the immune system must be fine-tuned in order to keep gut homeostasis. Thus, alterations affecting this dynamic interaction may trigger intestinal inflammation, such as inflammatory bowel disease (IBD) (3, 4).

IBD is a chronic, debilitating disorder from the gastrointestinal tract that comprises both Crohn’s disease (CD) and ulcerative colitis (UC). This disease poses a major clinical challenge since there is still lack of knowledge regarding its etiopathology, which in turn hampers the development of efficient therapies (5). Notwithstanding, it is well established that the development of an exacerbated immune response toward the gut microbiota, often enhanced by genetic susceptibility factors, is the main cause for the occurrence of this disease (5, 6). Throughout the years, several reports have demonstrated how commensal bacteria can be beneficial or detrimental for gut homeostasis, such as *Faecalibacterium prausnitzii* or *Bacteroides fragilis*, respectively (7, 8). However, some of these studies are circumscribed to the phylum level, therefore lacking the precision needed to pinpoint its specific players (9, 10). Moreover, it is unknown if interactions between different commensal bacteria may have any role on IBD development besides the impact that single microbes could have on disease progression. The findings collected in this work have shown that *A. muciniphila* and *P. distasonis* can play an important role in promoting protection against UC. In addition, this work revealed that the beneficial effect driven by *A. muciniphila* is dependent on an enrichment in type 3 innate lymphoid cells (ILC3) in the colon and by improving gut epithelial integrity. Moreover, *A. muciniphila* properties can be amplified by the co-colonization with *P. distasonis*, reinforcing the importance of studying the intricate interactions between microbiota players, rather than focusing on single-microbe probiotics to develop new microbiome-based therapies to tackle IBD.

## MATERIALS AND METHODS

### Mice

C57BL/6 wild-type mice used in this study were derived from two different animal facilities: one group was purchased from Charles River Laboratories (France), and the other group previously originated from the same commercial enterprise but was housed and bred at ICVS Animal Facilities, under specific pathogen-free conditions (four to six mice per cage). Rag2-knockout (Rag2-ko) mice used in this study were housed following the abovementioned conditions. Mice were euthanized by CO_2_ inhalation with efforts to minimize suffering.

### Colitis induction

Mice 7–9 weeks of age were given dextran sulfate sodium (DSS; 3% (wt/vol), molecular weight approximately 40,000 Da; TdB Consultancy) in the drinking water *ad libitum* for 7 days. Clinical signs of colitis were monitored daily and measured by the disease activity index (DAI, Table 1) using a graded score adapted from both Cardoso et al. and Gaifem et al. (11, 12). Mice were euthanized at the end of each experiment or earlier, if the symptoms of clinical disease reached one of these endpoints: more than 20% weight loss, diarrhea, or gross bleeding. For chronic colitis model, 2% DSS was given in the drinking water for 5 days in two subsequent phases with an interval of 3 weeks.

**TABLE 1:** Disease activity index scores^*a*^

Score	Weight loss	Stool consistency	Bleeding
0	No loss	Normal	No blood
1	1%–5%	Mild-soft	Brown color
2	6%–10%	Very soft	Reddish color
3	11%–15%	Diarrhea	Bloody stool
4	16%–20%		Gross bleeding
5	>20%		

^*a*^ The final score is obtained by the sum of each parameter.

### Histological analysis

Samples from colons were fixed in 4% paraformaldehyde, and 5-µm paraffin-embedded sections were stained with hematoxylin and eosin. Inflammation was assessed blindly by a pathologist using a graduated semiquantitative system as described below (Table 2) (11). Staining of colon sections with alcian blue/periodic acid-Schiff was performed to evaluate polysaccharide structures. The number of goblet cells were blindly evaluated for each experimental condition. Only intact crypts, cut longitudinally from crypt opening to bottom, were quantified. Images were captured using an Olympus BX61 microscope and recorded with a digital camera (DP70) using Cell^*P* software. Image analysis was performed using Fiji (ImageJ) software.

**TABLE 2:** Parameters for histological analysis of colitis severity^*a*^

Score	Epithelial hyperplasia and goblet depletion	Leukocyte infiltration in the lamina propria	Affected area	Markers of severe inflammation
0	None	None/rare	None	None
1	Minimal	Increased	One-third	Increased
2	Mild	Confluent	Two-thirds	Confluent
3	Marked	Transmural	All	Transmural

^*a*^ The final score is obtained by the sum of individual scores. Markers of severe inflammation included ulceration and crypt abscesses.

### Fluorescein isothiocyanate-dextran intestinal permeability assay

*In vivo* intestinal permeability was assessed by administration of fluorescein isothiocyanate (FITC) labeled dextran. Food and water were withdrawn for 8 hours. Mice were administered 44 mg/100 g body weight of FITC-labeled dextran (4 kDa, TdB Consultancy) by oral gavage. Serum was collected 4 hours later, and fluorescence intensity was measured by spectrophotofluorimetry (excitation: 485 nm; emission: 528 nm).

### RNA extraction, cDNA, and quantitative real-time PCR (qRT-PCR)

Total RNA was isolated from colonic samples using TripleXtractor (Grisp) with mechanical disruption of the tissues on ice, followed by conversion into cDNA by reverse transcription with Xpert cDNA synthesis kit (Grisp). qRT-PCR was performed using KAPA SYBR FAST Universal (Roche) on a Bio-Rad CFX6 Real-Time System C1000 Thermal Cycler (Bio-Rad). Specific oligonucleotides for mouse mucin-encoding genes *Muc1*, *Muc2*, and *Muc13*; for claudin-encoding genes *Cldn2*, *Cldn3*, *Cldn4*, and *Cldn7*; and for E-cadherin (*Cdh1)* are shown in Table 3. Expression levels were normalized to ubiquitin (*Ubq*) and relative expression was determined based on the ∆Ct method, as follows: 2^(housekeeping gene mRNA expression − target gene mRNA expression)^ × 100,000.

**TABLE 3:** List of primers used for PCR

Primer ID	Forward sequence (5′→3′)	Reverse sequence (5′→3′)
*Cdh1*	CACCTGGAGAGAGGCCATGT	TGGGAAACATGAGCAGCTCT
*Cldn2*	GGCTGTTAGGCACATCCAT	TGGCACCAACATAGGAACTC
*Cldn3*	AAGCCGAATGGACAAAGAA	CTGGCAAGTAGCTGCAGTG
*Cldn4*	CGCTACTCTTGCCATTACG	ACTCAGCACACCATGACTTG
*Cldn7*	AGGGTCTGCTCTGGTCCTT	GTACGCAGCTTTGCTTTCA
*Muc1*	CCCTATGAGGAGGTTTCGGC	AAGGGCATGAACAGCCTACC
*Muc2*	TCCTGACCAAGAGCGAACAC	ACAGCACGACAGTCTTCAGG
*Muc13*	CTGGCAGCTACATGAGCACT	GAACTACCCACGGTCACCAA
*Ubq*	TGGCTATTAATTATTCGGTCTGCAT	GCAAGTGGCTAGAGTGCAGAGTAA
Am	CAGCACGTGAAGGTGGGGAC	CCTTGCGGTTGGCTTCAGAT
Pd	TGCCTATCAGAGGGGGATAAC	GCAAATATTCCCATGCGGGAT

### Lamina propria leukocyte isolation and flow cytometry analysis

To isolate lamina propria leukocytes, colons were flushed with Ca- and Mg-free phosphate-buffered saline (PBS) with 25-mM HEPES (Gibco), 50-mM sodium bicarbonate (Sigma-Aldrich), and 5% fetal bovine serum (FBS, Gibco). Colon fragments of 0.5–1.0 cm were incubated in Ca- and Mg-free Hank’s balanced salt solution (Gibco) containing 1.3-mM EDTA (Sigma-Aldrich), 25-mM HEPES, 50-µg/mL penicillin/streptomycin (Gibco), and 2-mM L-glutamine (Gibco), under 200-rpm agitation at 37°C for 40 min, followed by an incubation in RPMI 1640 medium (Gibco) supplemented with 0.15-mg/mL collagenase D (Roche), 10% FBS, 25-mM HEPES, 50-µg/mL penicillin/streptomycin, and 2-mM L-glutamine for 40 min under 200-rpm agitation at 37°C. Tissue was dissociated and filtered through a 70-µm cell strainer (BD Biosciences). Cell suspension was centrifuged. The pellet was resuspended in 40% Percoll (GE Healthcare), laid over 80% Percoll, and centrifuged at 600 g for 20 min at 20°C. Cells retained in the interface were collected, washed in RPMI containing 2% FBS and recovered.

Cells were stimulated with 50 ng/mL phorbol myristate acetate, 500-ng/mL ionomycin calcium salt, and 10-µg/mL brefeldin A for 4 hours at 37°C (from Sigma-Aldrich). Cells were stained with eBioscience Fixable Viability Dye eFluor for viability control, followed by surface and intracellular staining using the eBioscience Foxp3/Transcription Factor Staining Buffer Set as per manufacturer’s instructions. Surface staining was performed with anti-mouse CD45 (clone 30-F11), CD90.2 (Thy1.2, clone 53–1.2), CD3 (clone 145–2C11), CD4 (clone GK1.5), CD19 (clone 6D5), CD11c (clone N418), and CD11b (clone M1/70) for 30 min at 4°C. Intracellular staining was performed for RORγT (clone B2D), interleukin (IL)-17A (clone TC11-18H10.1), and IL-22 (clone Poly5164), for 30 min at 4°C. All antibodies were purchased from BioLegend and eBioscience. The gating strategy is presented in Fig. S1. Cell analysis was performed on a BD LSRII (Becton Dickinson, USA). Data were analyzed using FlowJo software (Tree Star, USA).

### Cytokine quantification by ELISA

Colonic tissues were weighted and lysed using a homogenizer in ice-cold PBS containing protease inhibitors (Roche). Protein concentrations were quantified using the Pierce BCA protein assay kit (Bio-Rad). The levels of IL-10, IL-17A/F, and IL-22 were measured by ELISA using commercially available kits (BioLegend), according to the manufacturer’s instructions.

### Bacterial cultures

*Akkermansia muciniphila* (DMS 22959) from human origin was grown in modified yeast- and short-chain fatty acid-containing (mYCFA) culture medium (13) under anaerobic conditions in a vinyl anaerobic chamber (Coy Laboratory Products, USA). Bacteria were grown to an optical density (OD) of 1.0 before they were pelleted by centrifugation at 4°C for 10 min at 5,000 × *g*. Cells were resuspended in fresh mYCFA to reach an OD of 0.05 and then sealed anaerobically in 2-mL screw-cap tubes. The cultures were transported at ambient temperature by overnight express from Luxembourg to Portugal.

The mouse *A. muciniphila* was kindly provided by Eric Martens (University of Michigan, USA); this strain was isolated in Martens lab from wild-type C57BL/6 mice as described previously (14). Stocks of frozen murine *A. muciniphila* and *Parabacteroides distasonis* (DSM 29491; purchased from DSMZ, Germany) were inoculated at a concentration of 10% (vol/vol) in FEED medium [prepared as described in reference (15)], which simulates the fluids of the large intestine. The inoculated medium was kept at 37°C under anaerobic conditions (80:20, N_2_:CO_2_) for 24 hours. Afterward, bacteria were reinoculated in fresh FEED media (10% vol/vol) and kept under the same conditions until reaching an OD of 1.5 at 600 nm. Grown bacteria were plated in peptone yeast extract glucose agar (for *A. muciniphila*) or Columbia blood agar (for *P. distasonis*) in an anaerobic jar with Anaerogen 2.5 L (Thermo Scientific) for 48–72 hours to quantify the CFU/mL. New frozen stocks of bacteria were prepared in 25% glycerol and stored at −80°C. Bacterial cultures were incubated for 30–60 min at 37°C before administration.

### Microbiota modulation

#### Antibiotic treatment for microbiota depletion

C57BL/6 mice were given ampicillin (1 mg/mL), streptomycin (1 mg/mL), vancomycin (0.5 mg/mL), and neomycin sulfate (1 mg/mL) in drinking water for 4 weeks. All antibiotics were purchased from Sigma-Aldrich. Microbiota depletion was assessed throughout the treatment by aerobic and anaerobic culture of intestinal contents in Columbia agar plates with 5% sheep blood at 37°C. The number of CFUs were counted, and the number of bacteria per milligram of feces was calculated.

#### Fecal microbiota transplant

Fresh fecal contents from resistant mice were directly collected to a sterile 2-mL capped microtube, resuspended in ice-cold PBS (Gibco) and centrifuged (800 × *g* for 5 min) to remove residual clumps. The resuspended material (150 µL/day) was given by oral gavage to mice from the susceptible group. Fecal microbiota transplant (FMT) was performed after antibiotic treatment using a disease remission model as represented in Fig. 3A and C, respectively.

#### Bacterial administration

*A. muciniphila* and *P. distasonis* suspensions were prepared in sterile PBS with a final density of 2 × 10^9^ CFU/mL. According to the group, mice received daily 2 × 10^8^ CFU of each strain in 100 µL of PBS by oral gavage for 12 days. The control group received the same amount of PBS.

### Microbiome analysis and bacterial quantification

Genomic DNA from feces was extracted using the QIAamp Fast DNA Stool Mini Kit (Qiagen) according to manufacturer’s instructions plus an additional membrane disruption step using glass beads as previously described (16). After quantification of genomic DNA by spectrophotometry at 260 nm, 16S rRNA gene was amplified and sequenced using the MiSeq platform from Illumina and analyzed with mothur as previously described (17). Sequences were trimmed using the sliding-window technique, such that the minimum average quality score over a window of 20 bases never dropped below 30. Sequences were trimmed from the 3′-end until this criterion was met. Then, trimmed forward and reverse paired-end sequences were assembled using fastq-join (18), applying default parameters. Assembled paired-end sequences larger than 400 bp were kept for the subsequent analysis. Sequences were aligned to the 16S rRNA gene using the SILVA reference alignment as the template (19), and the Needleman-Wunsch algorithm with the default scoring options. Potentially chimeric sequences were removed using Uchime (20). To minimize the effect of sequencing errors in overestimating microbial diversity (21), rare abundance sequences that differ in 1% from a high abundance sequence were merged to the high abundance sequence using the pre.cluster option in mothur (22). Since different numbers of sequences per sample could lead to a different diversity [i.e., more operational taxonomic units (OTUs) could be obtained in those samples with higher coverage], we rarefied all samples to the number of sequences obtained in the sample with the lowest number of sequences (i.e., 27,287). Sequences were grouped into OTUs using Vsearch (23), with the abundance-based greedy (agc) clustering method. Sequences with distance-based similarity of 97% or greater were assigned to the same OTU. Shannon index was obtained at the OTU level with mothur.

Phylogenetic classification of sequences was performed for each sequence using the Bayesian classifier algorithm described by Wang and colleagues with a bootstrap cutoff of 60% (24). Classification was assigned to the genus level when possible; otherwise, the closest level of classification to the genus level was given, preceded by “unclassified; UC.”

Absolute abundance of bacteria was performed by quantification of bacterial copy number in stool DNA samples using specific primers for *A. muciniphila* (Am) (25) and *P. distasonis* (Pd) (26) (Table 3). Values were interpolated from a standard curve obtained by different copy numbers of the targeted sequence belonging to each bacterium. The targeted sequence for *A. muciniphila* or *P. distasonis* was cloned into pJET1.2 by CloneJET PCR Cloning Kit (Thermo Scientific) and used as a template for the quantitative PCR standard curve.

### Depletion of CD90^+^ ILCs

Depletion of CD90^+^ ILCs was performed in Rag2-ko mice by the administration of 250 µg of mAb anti-mouse Thy1.2 (CD90.2) (BioXCell), by intraperitoneal injection for a total of three times at 3-day intervals. The control group was treated with an isotype control rat IgG2b (IchorBio) in a similar manner. Intestinal lamina propria cells were collected 3 days after the final injection.

### Statistical analysis

For multiple group comparisons, *t*-test or one-way analysis of variance (ANOVA) test with a Tukey multiple-comparison post-test was performed, while for multiple group comparisons with repeated measures two-way ANOVA test with a Tukey multiple-comparison post-test was applied. For microbiota analysis data, a *t*-test was initially applied to identify bacterial genera whose relative abundance was increased in resistant and FMT-treated mice as compared to susceptible mice. Subsequently, we verified the obtained results by applying an approach recently developed specifically for studying microbiome data: analysis of composition of microbiomes with bias correction (ANCOM-BC) test (27). ANCOM-BC was applied using the R package ANCOMBC. Since the number of samples per group was not large (*n* = 5–6), a conservative variance estimate of the test statistic was used as recommended. To adjust for multiple hypothesis testing, for both ANCOMBC and *t*-test, we used the false discovery rate (FDR) approach by Benjamini and Hochberg implemented in the fdr.R package (28). Only taxa with at least 10 counts were included in the analysis. *q* values (FDR) lower than 0.05 were considered significant. PCoA analysis was performed using the Bray-Curtis distances (OTU level) between pairs of samples that were calculated using the package vegan from R. In order to analyze community-level differences in the microbiome among groups of samples, a non-parametric test, permutational multivariate ANOVA, was applied using the adonis function from the R vegan package. Images are representative of at least three independent experiments. Data are presented as mean ± standard deviation. Statistically significant values are *P < 0.05, **P < 0.01, ***P < 0.001, ****P < 0.0001.

## RESULTS

### Mice from different animal facilities display different susceptibility to chemically induced colitis

**Fig 1:**
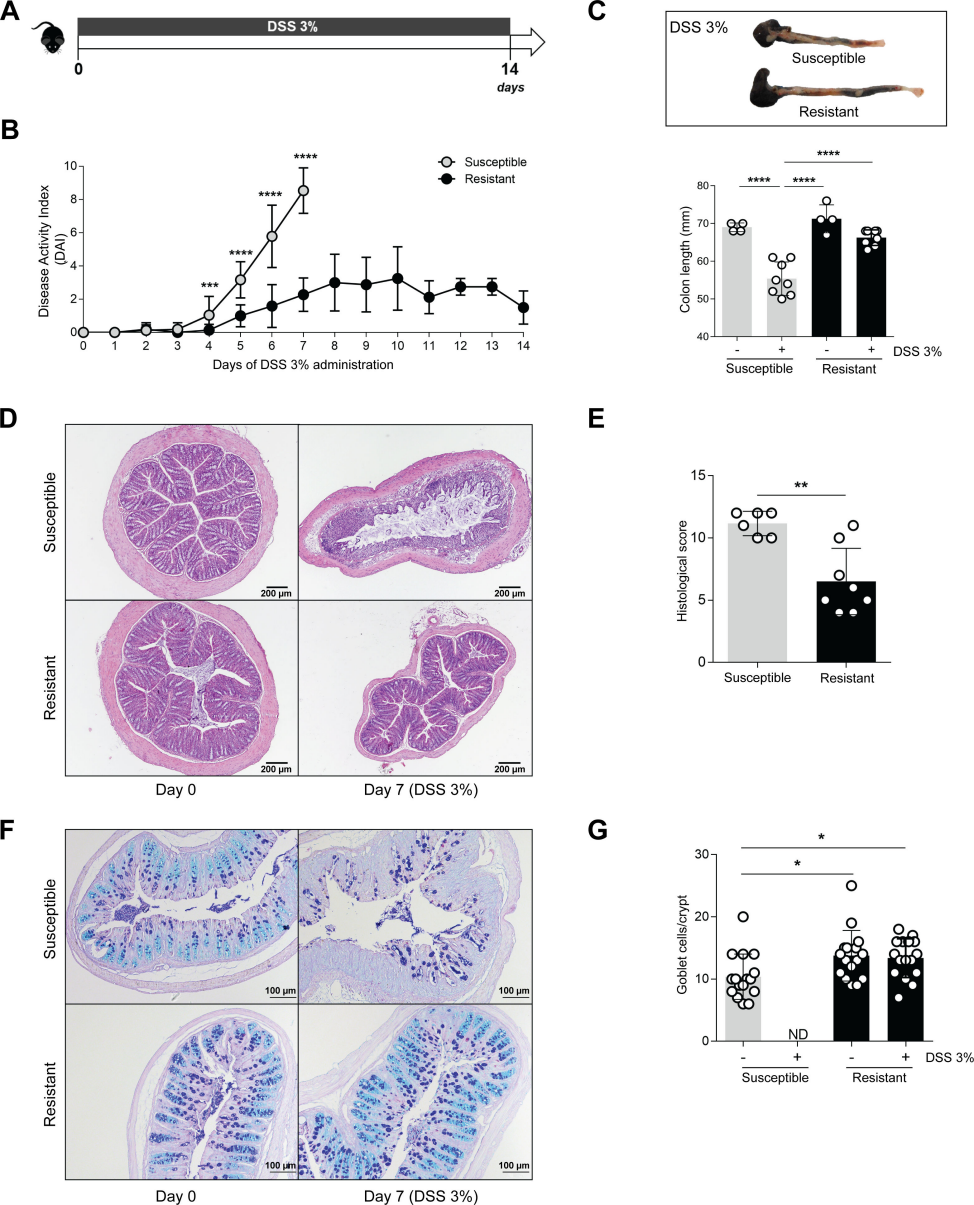
Mice from different animal facilities display distinct susceptibility to colitis development. (**A**) C57BL/6 mice from two different animal houses were administered with 3% DSS in the drinking water and were monitored daily. (**B**) Disease progression was assessed by scoring the DAI throughout the experiment. (**C**) Representative colons were imaged, and colon length was measured at day 7, after excision. (**D**) Histological analysis of hematoxylin and eosin staining of mice prior and after colitis induction. (**E**) Colitis scores were obtained by the histological evaluation of colon samples at day 7. (**F**) Alcian blue/periodic acid-Schiff staining of the colonic tissues for goblet cells and mucus analysis. (**G**) Quantification of goblet cell numbers per crypt. For susceptible mice at day 7, no intact crypts were found. Data are presented as mean ± standard deviation. Statistically significant values are *P < 0.05, **P < 0.01, ***P < 0.001, ****P < 0.0001. DAI, disease activity index; DSS, dextran sulfate sodium; ND, not detected.

To characterize the immune response associated with IBD development, we chemically induced colitis in wild-type C57BL/6 mice by DSS treatment for 7 days (Fig. 1A). All parameters associated with disease progression, such as weight loss, stool consistency, and presence of blood in stools, were monitored daily and scored according to the DAI. Unexpectedly, we observed that wild-type C57BL/6 mice did not display major clinical signs of disease development even when exposed to a prolonged treatment (Fig. 1B; from now on referred to as resistant). To study this unexpected phenotype, colitis was induced using a similar protocol on wild-type C57BL/6 mice housed in a different animal facility. These mice developed colitis, following the expected disease course, with an average DAI score of 8 (out of 10) at day 7 post-DSS administration (Fig. 1B; from now on referred to as susceptible). Upon examination, susceptible mice had shorter colons, consistent with significant colon pathology (Fig. 1C). Histological analysis of the colons, comprising ulceration, crypt shortening or ablation, and the presence of inflammatory infiltrates, showed a severe histopathology in the susceptible group when compared to those without severe disease progression (Fig. 1D and E). The total number of goblet cells were also different between susceptible and resistant mice under homeostatic conditions, with a massive reduction of goblet cells and mucus layer observed in susceptible mice only after DSS-induced colitis (Fig. 1F and G). Hence, despite being genetically identical and subjected to the same experimental protocol, mice from different animal facilities showed a different response to the induction of colitis.

### Resistant mice exhibit an upregulation of genes associated with epithelial barrier function and a distinct gut immunity

**Fig 2:**
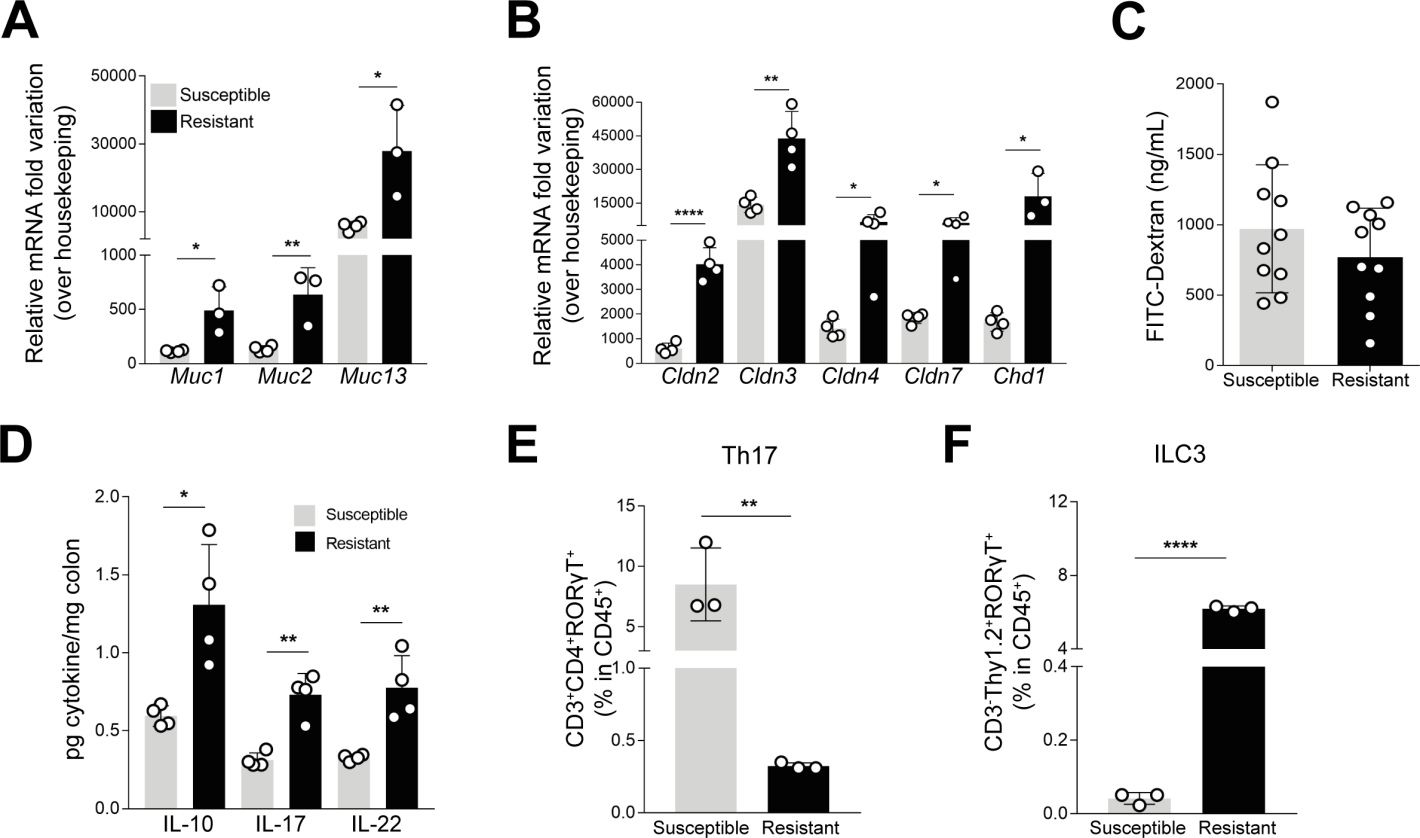
Resistant mice display alterations on epithelial barrier function and gut immunity. (**A and B**) Expression of *Muc1*, *Muc2*, *Muc13* (**A**), and *Cldn2*, *Cldn3*, *Cldn4*, *Cldn7*, and *Cdh1* (**B**) was analyzed by quantitative PCR in homeostatic conditions. (**C**) Intestinal permeability in homeostasis was measured after administration of FITC-dextran by oral gavage and quantified in the serum after 4 hours of administration. (**D**) The production of IL-10, IL-17, and IL-22 (pg cytokine/mg colon) was quantified in colonic extracts at homeostatic conditions. (**E and F**) Frequencies of Th17 cells (**E**) and ILC3 (**F**) in the gut of susceptible or resistant mice, under homeostatic conditions. For panels** E and F** , each dot corresponds to a pool of three mice. Data are presented as mean ± standard deviation. Statistically significant values are *P < 0.05, **P < 0.01; ****P < 0.0001. Th17, T helper 17.

Since the two groups of mice have the same genetic background but a divergent phenotype upon colitis induction, we hypothesized that alterations in the stability and function of the intestinal epithelial barrier could be associated with the observed phenotype. Alterations in the expression of tight and adherens junction proteins, such as claudins, were described in IBD patients, reinforcing the relevance of the integrity of the intestinal epithelial barrier in this pathology (29). Other important component of the epithelial barrier is the mucus layer constituted by mucin glycoproteins produced and secreted by goblet cells, which prevents the direct contact of luminal microorganisms (30). Under homeostatic conditions, the transcriptional levels of mucin-encoding genes (*Muc1*, *Muc2*, and *Muc13*), as well as E-cadherin- and claudin-encoding genes (*Cdh1*, *Cldn2*, *Cldn3*, *Cldn4*, and *Cldn7*), were significantly upregulated in resistant mice when compared to the susceptible group (Fig. 2A and B). These data suggest an hyperactivation of epithelial barrier-associated proteins in the resistant group, which may allow the epithelial barrier to sustain an inflammatory insult. Although exacerbated intestinal permeability is known to be a hallmark in IBD and is found in IBD patients (31), no significant differences were found in the intestinal permeability among the two groups of mice at homeostasis (Fig. 2C).

To determine whether the protective phenotype observed in resistant mice was driven by an altered gut immunity, the immune environment in the gut of both susceptible and resistant mice prior to colitis induction was evaluated considering the cytokine levels. IL-10 was found increased in resistant mice (Fig. 2D), which goes in line with the protective phenotype displayed by these mice. Additionally, resistant mice also presented higher levels of IL-17A and IL-22 when compared to susceptible mice (Fig. 2D). Both IL-17A and IL-22 can be produced by a myriad of immune cells present in the gut, namely, T helper 17 (Th17) cells and ILC3 and may have either proinflammatory or tissue-protective properties, depending on the context (32–34). We observed that susceptible and resistant mice have a divergent frequency of both ILC3 and Th17 cells in the gut at homeostatic conditions, with resistant mice displaying a significantly higher frequency of ILC3 and, inversely, lower frequency of Th17 cells, when compared to susceptible mice (Fig. 2E and F). Since ILC3 are one of the major producers of IL-22 in the gut and play a pivotal role in the maintenance of gut homeostasis (35), these results suggest that the increased frequency of ILC3 cells at steady state may be contributing to a healthier intestinal environment in resistant mice.

### Microbiome modulates protection against colitis development

**Fig 3:**
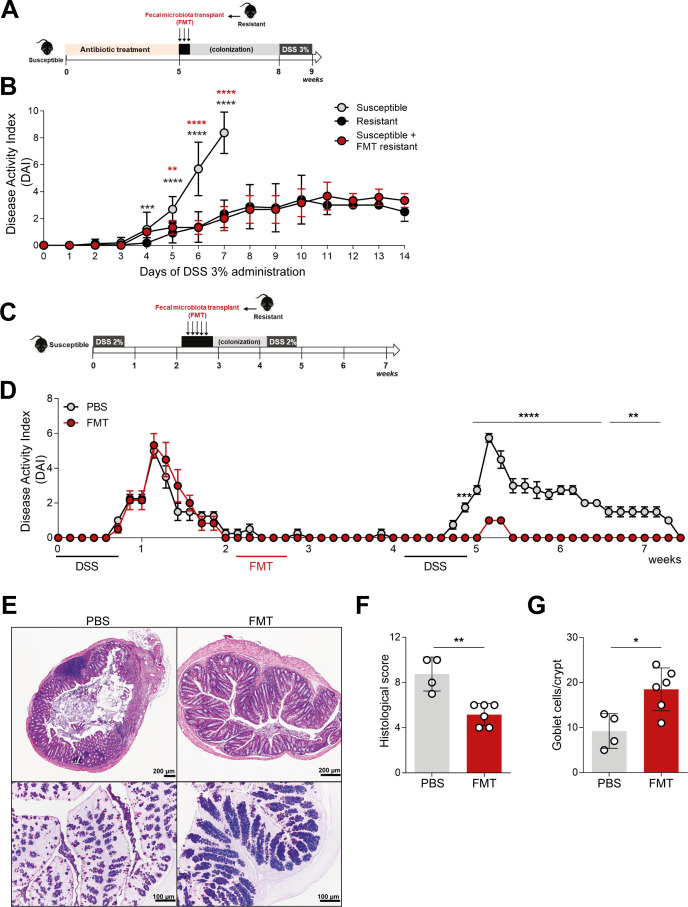
FMT from resistant mice is able to prevent the development of acute colitis and avoid relapse in chronic colitis in susceptible mice. (**A**) Susceptible mice were treated with antibiotic for 5 weeks and then received fecal contents from resistant mice by oral gavage for 3 days. After 3 weeks, to allow colonization, mice were challenged with 3% DSS for 7 days. (**B**) Disease progression was assessed by scoring the DAI throughout the experiment. Images are representative of at least three independent experiments; *n* = 5 per group. (**C**) Susceptible mice were treated with 2% DSS for 5 days. After remission, mice received FMT from resistant mice by oral gavage for 5 days. The control group was treated with the vehicle (PBS). Two weeks later, both groups were given 2% DSS as previously mentioned. (**D**) Disease progression was assessed by scoring the DAI throughout the experiment. (**E**) Histological analysis of hematoxylin and eosin and alcian blue/periodic acid-Schiff stainings of the colonic tissues from mice that received FMT or PBS at 7 weeks of treatment. (**F**) Colitis scores were obtained by the histological evaluation of colon samples at week 7. (**G**) Quantification of goblet cell numbers per crypt. Images are representative of at least three independent experiments; *n* = 5 per group. Data are presented as mean ± standard deviation. Statistically significant values are *P < 0.05, **P < 0.01, ***P < 0.001, ****P < 0.0001. FMT, fecal microbiota transplant.

To determine if the protective phenotype upon colitis induction was driven by a distinctive microbiota composition, an FMT, with fecal contents from resistant mice, was performed into antibiotic-induced microbiota-depleted susceptible mice (Fig. 3A). Susceptible mice were previously treated with a mixture of antibiotics for 5 weeks to deplete the native gut microbiota. The efficacy of the microbiota depletion was assessed throughout the antibiotic treatment (Fig. S2). After this, the susceptible group received a fecal suspension from resistant mice for 3 consecutive days. Three weeks after FMT, mice were submitted to DSS treatment for colitis induction, and the DAI was compared with susceptible and resistant mice without FMT. A clear protection against colitis induction was observed (Fig. 3B). These data, mirroring the resistant group as reflected by their similar DAI, support the key role of gut microbiome in the protective phenotype against colitis development.

In order to understand the protective effect in a relapse-remission model of colitis, disease was induced in susceptible mice and, upon remission of the disease, mice received FMT from the resistant mice by oral gavage during five days. No antibiotic treatment was administered in this setting. Two weeks after FMT, mice were again challenged with colitis (Fig. 3C). Mice treated with fecal contents from resistant mice displayed only mild symptoms of colitis, contrary to the control group that only received the vehicle (PBS) (Fig. 3D). FMT-treated mice displayed significantly fewer signs of pathology than the control group as well as higher amounts of goblet cells per crypt (Fig. 3E through G).

### *Akkermansia* and *Parabacteroides* spp. are significantly increased in the gut microbiota community of mice protected against colitis induction

**Fig 4:**
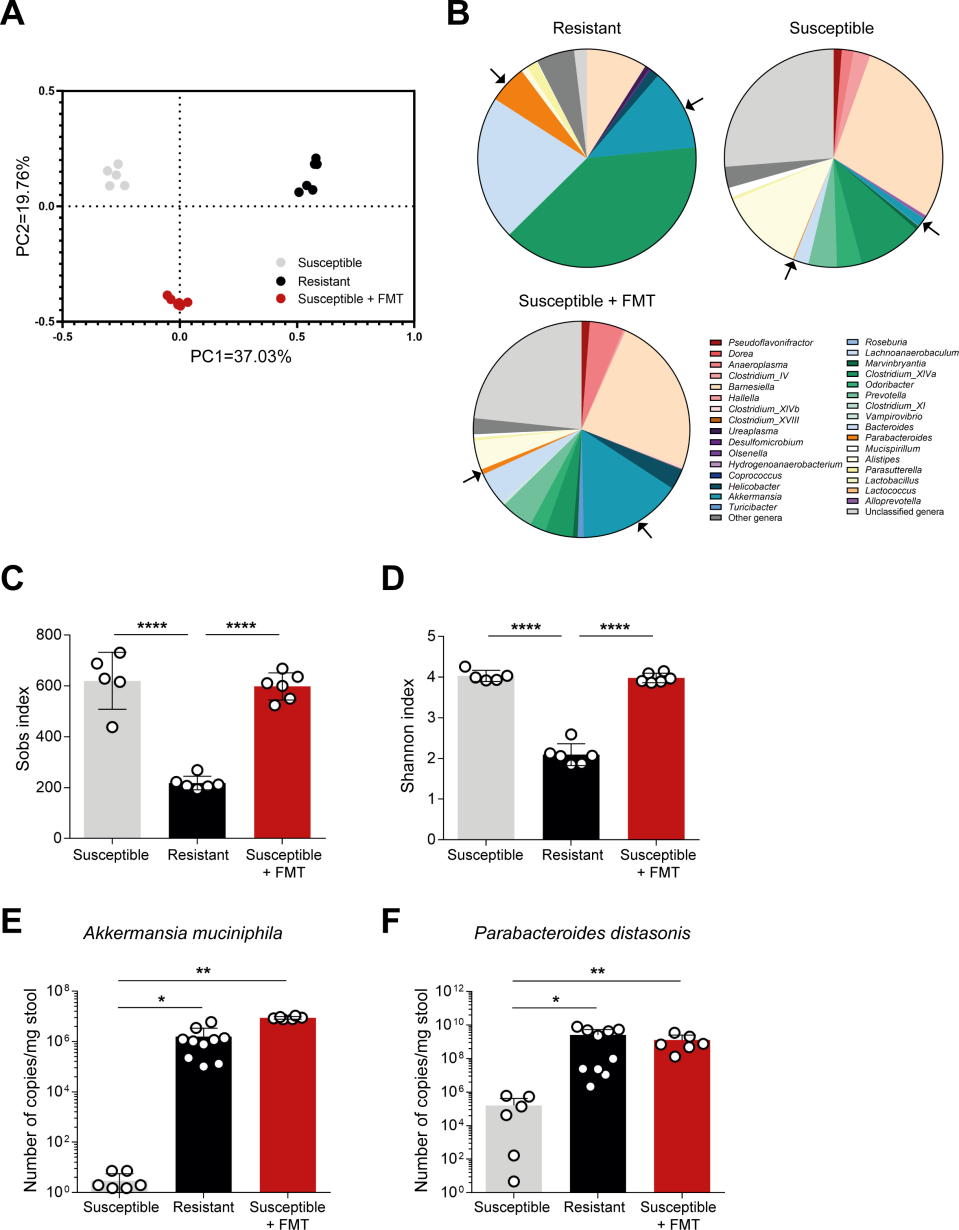
16S rRNA sequencing of fecal samples from resistant and susceptible mice revealed a distinct microbiota composition. (**A**) Principal coordinate analysis of susceptible, resistant, and susceptible + FMT mice regarding intestinal microbiota composition. Resistant vs susceptible: *P* = 0.001; resistant vs susceptible + FMT: *P* = 0.005; susceptible vs susceptible + FMT: *P* = 0.006. (**B**) Relative abundance of bacterial genera identified from DNA from stool samples of susceptible, resistant mice and susceptible + FMT mice; *A. muciniphila* and *P. distasonis* are marked by purple and beige arrows, respectively. (**C**) Number of operational taxonomic units and (**D**) diversity of species found in susceptible, resistant, and susceptible + FMT mice. (**E**) Absolute abundance quantification of *A. muciniphila* and (**F**) *P. distasonis* in susceptible, resistant, and susceptible + FMT mice. Images are representative of at least three independent experiments; *n* = 5/6 per group. Data are presented as mean ± standard deviation. Statistically significant values are *P < 0.05, **P < 0.01, ****P < 0.0001.

16S rRNA gene analysis of the gut microbiota composition was performed on the stools of mice, both susceptible and resistant, in homeostatic conditions. A distinct microbiota signature was found when comparing resistant and susceptible mice, with these clustering separately in an unsupervised multivariate analysis (Fig. 4A and B). The resistant group of mice presents a significant reduction of richness (Fig. 4C) and species diversity (Fig. 4D) which was unexpected since the decrease in number and diversity is often associated with disease (36). To pinpoint which bacterial species could be underlying the protective phenotype against colitis, susceptible mice that received FMT from resistant mice were also included in the microbiota analysis. No major alterations were observed in the susceptible group before or after the FMT regarding number and diversity of species. Yet, very clear shifts in the relative abundance of a limited number of genera, such as *Akkermansia*, suggest that minority populations could be responsible for the protective phenotype (Fig. 4A through D). Indeed, from the 129 genera identified, 6 were found to be significantly different between resistant and susceptible mice, while 7 were found significantly different between susceptible mice and susceptible mice that received FMT (*P* < 0.05, FDR < 0.05; Table 4). These hits were also validated using ANCOM-BC test (Tables S1 and S2). *Akkermansia* and *Parabacteroides* genera were pinpointed as our candidates, given their significantly increased number of copies when a resistant phenotype was observed (for *Akkermansia*, the relative abundances were 1.29%, 12.07%, and 15.55% in susceptible, resistant, and susceptible + FMT groups, respectively; for *Parabacteroides*, the relative abundances were 0.14%, 5.66%, and 0.78% in susceptible, resistant, and susceptible + FMT groups, respectively). A Basic Local Alignment Search Tool analysis of the 16S RNA gene sequences pinpointed *Akkermansia muciniphila* and *Parabacteroides distasonis* as the representative species for each genus.

**TABLE 4:** Most significant hits found in the 16S rRNA sequencing analysis (*P* value < 0.05, FDR <0.05)^*a*^^,^^*b*^

Increased in resistant mice (vs susceptible)				Increased in susceptible + FMT mice (vs susceptible)			
Genus	Log2FC	*P* value	Adjusted *P* value	Genus	Log2FC	*P* value	Adjusted *P* value
*Clostridium_*XlVa	2.0492	0.00002002	0.00138125	*Akkermansia*	3.5844	0.0000225	0.00153222
*Akkermansia*	3.2186	0.00131297	0.01132440	*Parabacteroides*	2.4413	0.00028237	0.00860324
*Parabacteroides*	5.2930	0.00204649	0.01283708	*Clostridium_*XI	4.3576	0.00037956	0.00860324
*Lactococcus*	2.8413	0.00559808	0.02425864	*Bacteroides*	1.1833	0.00085129	0.01447191
*Ureaplasma*	7.4717	0.00755823	0.03067753	*Olsenella*	1.6756	0.00342211	0.02908795
*Bacteroides*	3.2818	0.00835472	0.03202644	*Clostridium_*XlVb	1.5458	0.00918029	0.04855569
				*Coprococcus*	2.0000	0.00928271	0.04855569

^*a*^ FC, fold change; FDR, false discovery rate.

^*b*^ Comparison is made by resistant vs susceptible mice and susceptible + FMT vs susceptible mice.

To confirm that *A. muciniphila* and *P. distasonis* were increased in mice that showed protection against colitis induction, we examined the absolute abundance of these bacteria. As expected, *A. muciniphila* and *P. distasonis* were significantly increased in both resistant and susceptible mice after FMT when compared to susceptible mice (Fig. 4E and F). The abundance of *A. muciniphila* and *P. distasonis* in resistant mice is, on average, 500,000 and 15,000 times higher, respectively, than in susceptible mice, while for susceptible mice, after FMT, the abundance of *A. muciniphila* and *P. distasonis* was 3,000,000 and 7,000 times higher, respectively, than before receiving FMT. Overall, this result confirms the significant representation of these two species in the intestinal microbiota of mice protected against colitis, pointing to a possible effect of these bacteria, alone or in combination, in creating a very particular immunological environment that allows the intestine to sustain an insult.

### *Akkermansia muciniphila* and *Parabacteroides distasonis* act synergistically toward a decreased colitis severity

**Fig 5:**
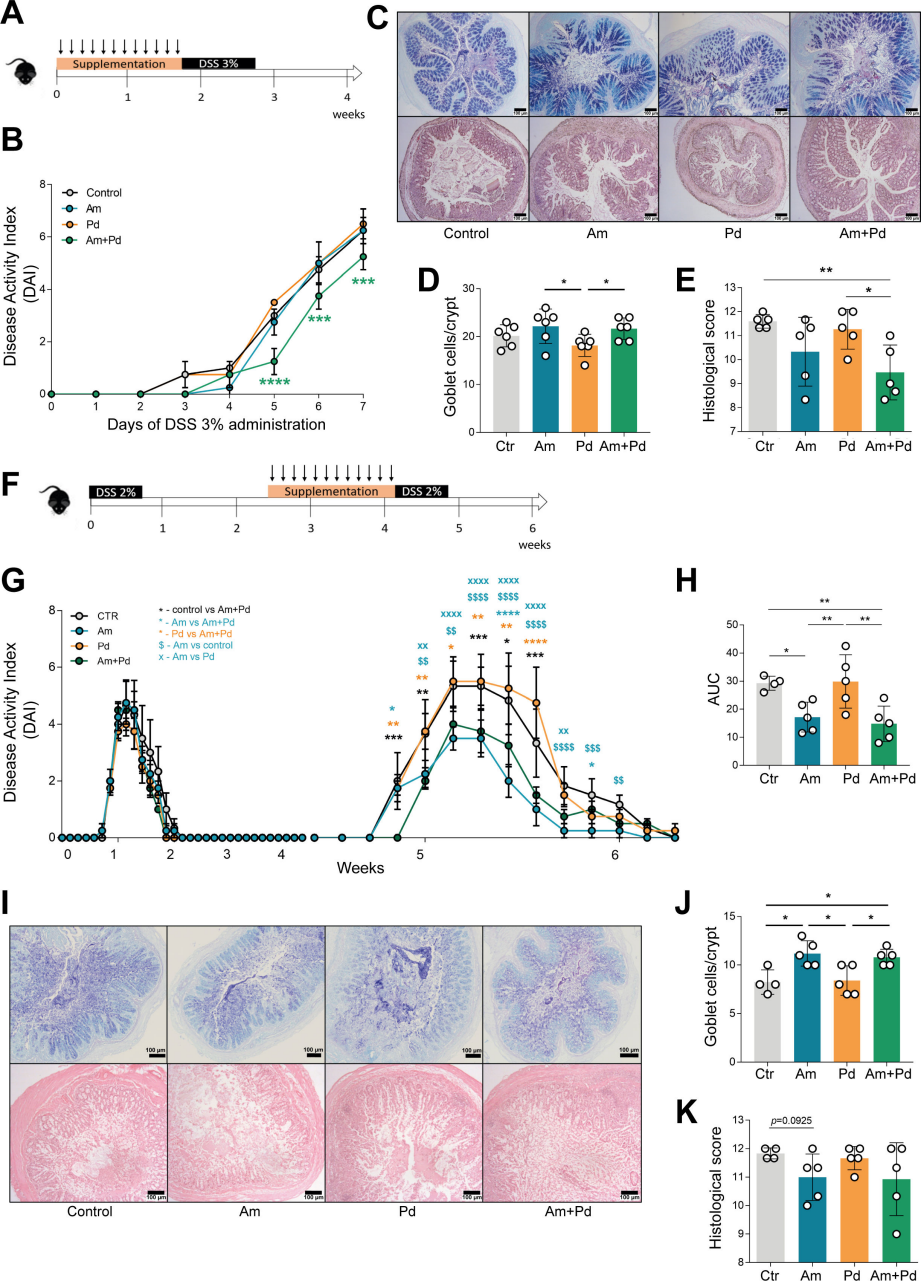
The combination of *Akkermansia muciniphila* and *Parabacteroides distasonis* attenuates colitis development. (**A**) Mice were supplemented with Am, Pd, or a combination of both (Am + Pd) for 12 days by daily oral gavage, followed by administration of 3% DSS for 7 days. (**B**) Disease progression was assessed by scoring the DAI throughout the experiment. (**C–E**) Quantification of goblet cell numbers per crypt after colitis induction, as well as colitis scores obtained by the histological evaluation of colon samples after DSS treatment. *N* = 5 per group. (**F**) A relapse-remission experiment was performed in which susceptible mice were subjected to colitis induction with 2% DSS for 5 days. After recovery, mice were supplemented with Am, Pd or the combination of both (Am + Pd) during 12 days by daily oral gavage, followed by a second cycle of colitis induction. Control mice (unsupplemented) received PBS as vehicle. (**G**) Disease progression was assessed by scoring the DAI. * in black corresponds to comparison between control and Am + Pd. * in blue corresponds to comparison between Am and Am + Pd. * in yellow corresponds to comparison between Pd and Am + Pd. $ in blue corresponds to comparison between control and Am. x corresponds to comparison between Am and Pd. (**H**) AUC was calculated based on the disease course upon colitis induction. (**I–K**) Quantification of goblet cell numbers per crypt after colitis induction, as well as colitis scores obtained by the histological evaluation of colon samples after DSS treatment. n = 5 per group. Data are presented as mean ± standard deviation. Statistically significant values are *P < 0.05, **P < 0.01, ***P < 0.001, ****P < 0.0001. Am, *Akkermansia muciniphila*; AUC, area under the curve; Ctr, control; Pd, *Parabacteroides distasonis*.

To assess the protective properties of *A. muciniphila* and *P. distasonis* in controlling inflammation in the chemically induced colitis, susceptible mice were supplemented with bacteria for 12 days before colitis induction with 3% DSS (Fig. 5A). Administration of *A. muciniphila* or *P. distasonis* alone was not able to reduce colitis. In contrast, administration of *P. distasonis*, in addition to *A. muciniphila*, significantly reduced the level of colitis as compared with control mice (Fig. 5B). The protective effect of *A. muciniphila* was also maintained when a murine-origin isolated strain was used (Fig. S3). Although no major alterations were found in terms of goblet cell numbers (Fig. 5C and D), the histological analysis showed that mice supplemented with both *A. muciniphila* and *P. distasonis* (Am + Pd) displayed less architectural damage and inflammation, with a histological score lower than those of the control group and Pd (Fig. 5C through E). This points toward a beneficial effect of the combination of *A. muciniphila* with *P. distasonis* in controlling the inflammation associated with acute colitis.

The protective effect of the combination of *A. muciniphila* with *P. distasonis* was also evaluated in a relapse-remission chronic model of colitis, in which susceptible mice were supplemented with these bacteria by oral gavage during 12 days between two cycles of DSS induction (Fig. 5F). We observed that supplementation with Am, alone or in combination with *P. distasonis* (Am + Pd), was able to partially protect the mice from the second cycle of DSS-induced colitis. Conversely the supplementation of Pd alone was insufficient (Fig. 5G). The best performance overall was indeed for *A. muciniphila*, alone or in combination with *P. distasonis*, as it is shown by the decreased area under the curve (Fig. 5H). *A. muciniphila* and the combination of both bacteria also led to an increased goblet cell count when compared to the control and Pd groups (Fig. 5I and J). No statistical differences were found in the histological analysis, despite the tendency of *A. muciniphila* and *A. muciniphila* with *P. distasonis* toward a lower score of pathology (Fig. 5I through K). Our results suggest that the combination of both bacteria is more effective in inducing protection against acute inflammatory events, with *A. muciniphila* supplementation standing out during chronic inflammation.

### Supplementation with *Akkermansia muciniphila* shapes gut immunity by promoting ILC3 population in the gut

**Fig 6:**
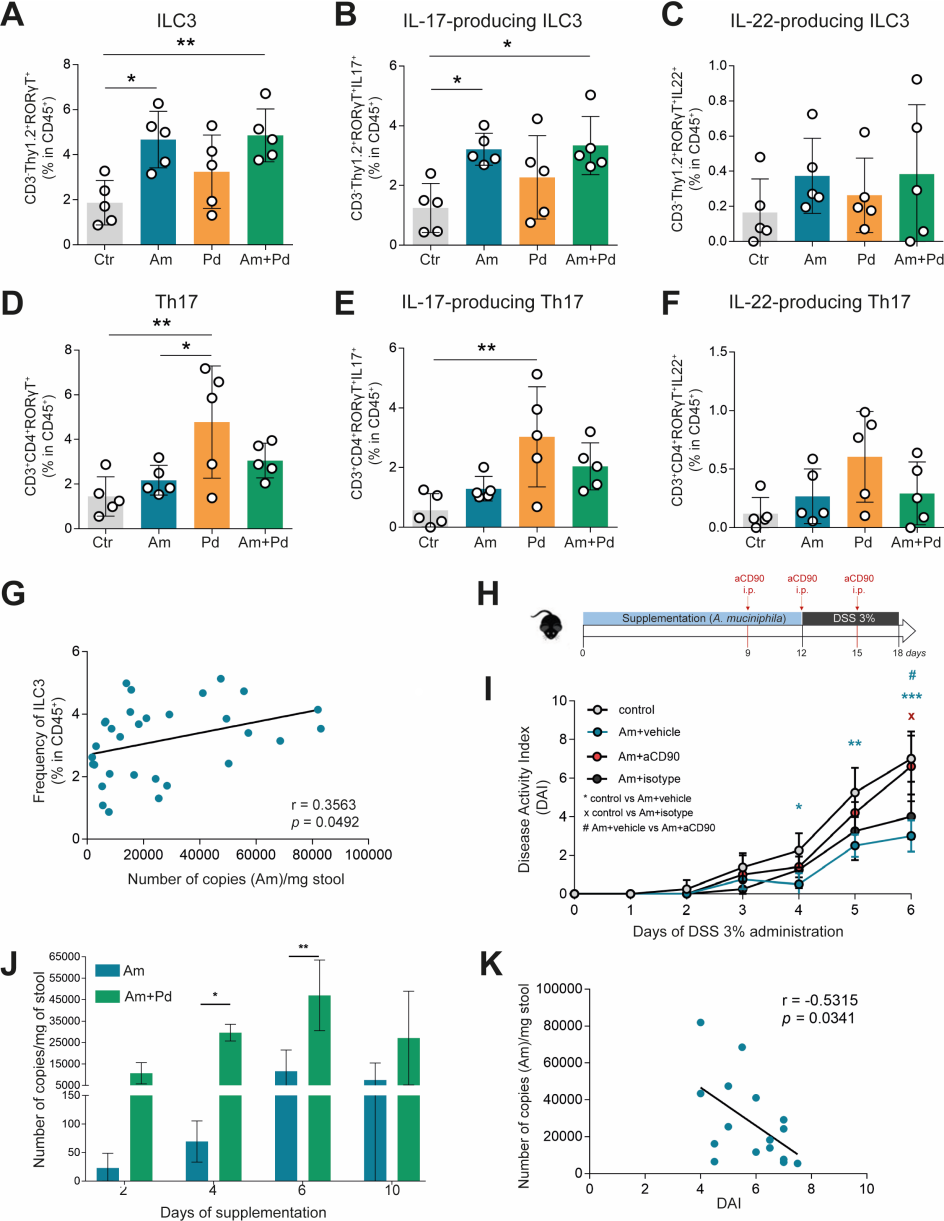
Supplementation with *Akkermansia muciniphila* leads to increased frequencies of ILC3 in the colon and tissue integrity. (**A–F**) Frequencies of ILC3 (**A**), IL-17-producing ILC3 (**B**), IL-22-producing ILC3 (**C**), Th17 cells (**D**), IL-17-producing Th17 (**E**), and IL-22-producing Th17 (**F**) in the colon of control mice and mice supplemented with Am, Pd, or the combination of both (Am + Pd). (**G**) Correlation between the number of copies of Am with the frequencies of ILC3 in the colonic tissue. (**H and I**) Mice were supplemented with Am for 12 days by oral gavage and treated with anti-CD90 monoclonal antibody (Am + aCD90) at days −3, 0, and 3 of 3% DSS treatment or no antibody treatment (Am + vehicle). As experimental controls, an isotype control (Am + isotype) was given instead of anti-CD90, or no antibody treatment was performed (Am + vehicle). Mice were given 3% DSS, and colitis development was assessed by DAI. The control group is related to mice that were not supplemented or treated. (**J**) Absolute abundance of *Akkermansia muciniphila*, alone (Am) or in combination with *Parabacteroides distasonis* (Am + Pd) in the colon of supplemented mice. (**K**) Correlation between the number of copies of Am with the DAI. Data are presented as mean ± standard deviation. Statistically significant values are *P < 0.05, **P < 0.01, ***P < 0.001.

It is known that microbiota interact with the immune system, either directly or by producing signals that, in turn, will regulate the response of immune populations, such as ILC3 (37). To understand whether supplementation with *A. muciniphila* is indeed interfering with the gut immune response, the immune profile was characterized upon bacterial supplementation (homeostatic conditions). Mice supplemented with *A. muciniphila*, alone or in combination with *P. distasonis*, showed improved epithelial barrier integrity (Fig. S4) and increased ILC3 frequencies when compared with the control group (Fig. 6A). This is accompanied with an increase in IL-17-producing ILC3 (Fig. 6B) and a tendency, although not significant, in IL-22-producing ILC3 (Fig. 6C). On the other hand, supplementation with Pd leads to an increase in Th17 and IL-17-producing Th17 frequency when compared with the control and Am treatment (Fig. 6D and E). No major alterations were found in the frequencies of IL-22-producing Th17 cells (Fig. 6F). Indeed, the number of copies of *A. muciniphila* was found to be positively correlated with ILC3 levels in the gut (Fig. 6G). Th17 and ILC3 have a crucial, yet dichotomic profile in managing homeostasis and inflammation, with Th17 being highly involved in intestinal inflammation and ILC3 being an important player in promoting gut homeostasis (38, 39). This, together with observations in which *A. muciniphila* and *A. muciniphila* with *P. distasonis* exerted a beneficial impact on colitis development, suggests that *A. muciniphila* can shape gut immunity toward a more homeostatic immune profile, conferring some degree of protection against an inflammatory event. To confirm that *A. muciniphila*’s protective effect was due to increased ILC3 frequencies in the gut, we supplemented Rag2-ko mice, which have defective T- and B-cell populations, with *A. muciniphila*, and then CD90^+^ ILCs were depleted in one of the experimental groups (Fig. S5; Fig. 6H). As expected, mice treated with *A. muciniphila* (Am + vehicle) exhibited reduced susceptibility to colitis compared to non-supplemented mice (control). Importantly, this protective effect of *A. muciniphila* supplementation is lost upon CD90^+^ ILC depletion (Am + aCD90), demonstrating that *A. muciniphila* contributed to gut immunity by promoting ILC in the colon, thus leading to protection against colitis (Fig. 6I).

We observed that *A. muciniphila* had a protective action against colitis development, benefiting in the acute model by the presence of *P. distasonis.* In this sense, we questioned if *P. distasonis* had any positive effect on the colonization by *A. muciniphila*. Although *A. muciniphila* abundance increases along the time due to the supplementation, it is strikingly increased when administered in combination with *P. distasonis* (Fig. 6J). This suggested a symbiotic relationship between *A. muciniphila* and *P. distasonis* that may be the responsible factor for the increased beneficial effect observed when the two bacterial species are combined. In support of this, we found that the quantity of *A. muciniphila* was inversely correlated with the DAI (Fig. 6K), which supports the protective effect observed in supplemented mice.

Altogether, these results suggest that *A. muciniphila* exerts a protective effect on colitis development by promoting an increase in ILC3 in the gut, thus controlling inflammation, and *P. distasonis* positively contributes to its colonization.

## DISCUSSION

Alterations in composition of gut microbiota are known to occur in several human diseases (36, 40, 41). A profound impairment in gut microbial composition occurs in IBD that largely contributes to the development and/or the severity of the disease (36, 42). A growing body of evidence has been stating the great involvement of microbiota to dictate protection or susceptibility to develop IBD, leading to an increased attention on the development of novel microbiota-derived therapies to tackle a disease which current treatments are not equally effective for all patients. Targeting the intestinal microbiota itself is not new; several approaches have already been described, such as fecal microbiota transplantation to control *Clostridium difficile* infection (43) or the use of specific strains of probiotics (9, 44). Nevertheless, the major aim in using microbiota modulation as a reliable strategy to treat or prevent IBD is the need for an effective immunomodulatory effect either locally or at the periphery. For this, a particular combination of players is pivotal for the ability to suppress proinflammatory strains and to promote those that create a more tolerant immune environment. Thus, it is important to identify protective commensal bacteria and to understand their ability to modulate immune cell populations to move forward as a potential therapeutic approach for IBD.

Here, we have revealed that the enrichment in *A. muciniphila* and *P. distasonis* bacteria in the gut exerts protection in both acute and chronic models of colitis induction. This microbial signature is concomitant with an increase in ILC3 frequency in the gut and with increased gut epithelial integrity, creating a balanced intestinal environment that is more prone to control inflammation and protect against severe forms of colitis. While a potential protective effect has been placing *A. muciniphila* as a promising probiotic to tackle intestinal inflammation (45), conflicting reports in murine experimental models (46, 47) and IBD patients (48, 49) have been disputing the *P. distasonis* role, being associated with enhanced or attenuated colitis development (50). *A. muciniphila* is a strict anaerobe mucin-degrading bacterium that represents around 1%–5% of the human intestinal microbial composition (51). It is able to degrade mucin, leading to the production of the short-chain fatty acids (SCFAs) propionate and acetate, which contribute to the regulation of host biological processes, including gut immune response (52, 53). The beneficial effect of *A. muciniphila* has been broadly described in the literature. For instance, it has been shown that *A. muciniphila* emerged as a promising candidate to treat or prevent obesity-related metabolic disorders (54, 55), with the ability to be associated with microbial species known to be related to health (56). Indeed, it was recently shown that daily oral administration of the pasteurized form of *A. muciniphila* alleviated diet-induced obesity, likely by a reduction of carbohydrate absorption and enhanced intestinal epithelial turnover (57). Additionally, *A. muciniphila* has been pinpointed as a promising player to induce intestinal protection. Lower abundances of *A. muciniphila* were described in IBD patients (58), which were correlated with higher inflammatory scores (59). A recent study showed that *A. muciniphila* can be beneficial also in a model of acute colitis with a mucosal pathogen, *Citrobacter rodentium*; however, this beneficial effect was context dependent, as when the mice were fed a fiber-deficient diet, *A. muciniphila* rather promoted pathogen susceptibility (60). A similar context-dependent detrimental effect of *A. muciniphila* was observed as it exacerbated food allergy in fiber-deprived mice (61).

Despite several studies pointing toward a protective role of *A. muciniphila* in controlling intestinal inflammation, the exact mechanisms by which this bacterium hampers disease progression are still not fully understood. *A. muciniphila* supplementation has been shown to reduce NLRP3 inflammasome in DSS-induced acute colitis (62) and to be partially responsible for the beneficial role of metformin in a mouse model of UC (63). It was also described that *A. muciniphila* administration was able to reduce inflammation driven by DSS treatment in mice, not only by regulating the colonic and serum levels of inflammatory cytokines such as tumor necrosis factor alpha and IL-6, but also by imposing alterations on the gut microbiota community and rescuing microbiota dysbiosis derived from DSS administration (64). Our work has revealed that the administration of *A. muciniphila* alone or in combination with *P. distasonis* leads to an increase of ILC3 and IL-17^+^-ILC3 that is associated with control of colitis in mice, reinforcing the interaction between microorganisms in the gut and their contribution to a protective immune response. ILC3 are particularly relevant in the regulation of intestinal homeostasis by the production of IL-17, IL-22, and granulocyte-macrophage colony-stimulating factor at steady state (39). It is known that both UC and CD patients display alterations in ILC3 populations, namely, in their ability to produce IL-22, which may be linked to the enhanced epithelial damage found in these patients (65, 66). How the immune subsets respond to the divergent environmental cues, such as microbiota composition, needs to be further clarified. For instance, it is described that SCFAs produced by intestinal microbiota have the ability to induce ILC3 and also IL-22 production via AKT-STAT3 signaling pathway (67). This reinforces the need for studying the impact of microbiota-derived metabolites in the modulation of intestinal homeostasis. Anyway, we have shown that the increased frequency of ILC3 in the gut may be largely shaped by the amount of *A. muciniphila* present in the gut, in a dose-dependent manner. We also demonstrated that the protective properties of *A. muciniphila* are intricately associated with this increase in ILC3 frequency in the gut. Other reports investigating the interaction between microorganisms and ILC3 have shown that several mechanisms can induce ILC3 function, particularly IL-22 production. These include the promotion of ILC3 through IL-23 and IL-1β produced by dendritic cells, as well as ILC3 activation via NKp44 by enteric bacteria (68). It remains to be determined in future studies whether *A. muciniphila* promotes an increase in ILC3 in the gut through direct contact or via mediation by other factors. In this sense, this work unveils a novel interaction between microbiota and immune response that needs to be explored in the future, in order to disclose a protective strategy to avoid or control gut inflammation.

It was previously demonstrated that *A. muciniphila* and *Parabacteroides* can synergistically be involved in the prevention of epilepsy by the decrease in gammaglutamylation of amino acids and the increase of hippocampal GABA/glutamate ratios, subsequently preventing seizures (69). This is particularly relevant since previous studies focusing on the effects of each of these bacteria in intestinal inflammation have not addressed the possibility of the combination of both in promoting intestinal protection, which supports the novelty of this work. In fact, we observed the tolerant effect of *A. muciniphila* in controlling colitis induction in mice, but interestingly, we found that this effect was even more pronounced when combined with *P. distasonis*. While we observed an advantageous and accelerated *A. muciniphila* gut colonization in the presence of the aerotolerant *P. distasonis*, the mechanisms by which this symbiotic interaction is established are an important topic for future studies. Yet, the metabolism of these bacteria may be the key to answer this question. It is described that *P. distasonis* is able to synthesize acetate and succinate (70). It is also known that *A. muciniphila* is a major propionate producer, mainly via mucin fermentation (71, 72). It is also described that the production of propionate by *A. muciniphila* can be promoted by vitamin B_12_, which is used as a co-factor in the conversion of succinate to propionate via methylmalonyl-CoA synthase (73). Thus, we can hypothesize that this synergistic effect of the combination of *A. muciniphila* and *P. distasonis* may be explained by a commensal feeding mechanism in which *P. distasonis* may be providing an extra source to *A. muciniphila* for propionate production, benefiting its metabolism and colonization.

The *A. muciniphila* and *P. distasonis* co-supplementation had also a positive impact on the intestinal epithelial barrier. Mice enriched with *A. muciniphila* and *P. distasonis* displayed an upregulation of genes involved in the maintenance of epithelial barrier stability, such as mucins and claudins, increased number of goblet cells, and decreased histological and disease score upon colitis induction when compared with susceptible mice. These results point out that the presence of these bacteria prepares the epithelial barrier to better sustain an inflammatory insult. This goes in line with previous data that highlight the capacity of *A. muciniphila* to promote intestinal epithelial barrier integrity by its capacity to strengthen enterocyte monolayer *in vitro* (74) and by releasing extracellular vesicles with anti-inflammatory properties that promote gut protection (75).

Bacterial composition in the gut can rapidly fluctuate due to environmental cues, imposing a huge challenge in the identification of specific beneficial microbes to intestinal health. Within this work, we pinpointed two specific bacterial species that, when combined, are able to promote intestinal protection by shaping gut immunity toward a more tolerant, homeostatic environment. It would be important to dissect the mechanisms underlying the protective effect of these combined microbes with disease severity and gut immunity. The observed protective phenotype observed is directly dependent on the levels of these bacteria in the gut, which rapidly decrease if the supplementation is stopped. In this sense and based on the local and systemic protective effect described for *A. muciniphila*, it would be crucial to study the stability of the supplementation with a mixture containing these bacteria and its ecologic and functional impact in other microbial populations to assess its full potential as a probiotic. In addition, to ensure if bacterial supplementation as a probiotic can have a long-lasting effect or, at least, can be more effective in controlling the inflammatory processes associated with IBD is a key factor that must be thoroughly studied. Overall, and despite the need for more complementary studies, this work provided a solid contribution in supporting the role of the gut microbiota in IBD development and prevention, undoubtedly a major topic to explore new strategies to tackle IBD.

## SUPPLEMENTAL MATERIAL

Supplemental Figures mbio.00078-24-s0001.pdf Fig. S1-S5.

Supplemental Tables mbio.00078-24-s0002.docx Tables S1 and S2.

ASM does not own the copyrights to Supplemental Material that may be linked to, or accessed through, an article. The authors have granted ASM a non-exclusive, world-wide license to publish the Supplemental Material files. Please contact the corresponding author directly for reuse.
