# Supplementary material for: Akkermansia muciniphila and Parabacteroides distasonis synergistically protect from colitis by promoting ILC3 in the gut
Source: mBio. 2024 Mar 12;15(4):e00078-24. doi: 10.1128/mbio.00078-24 (PMC11210198; doi:10.1128/mbio.00078-24)
Supplement: Supplemental Figures — Fig. S1-S5. [file mbio.00078-24-s0001.pdf]

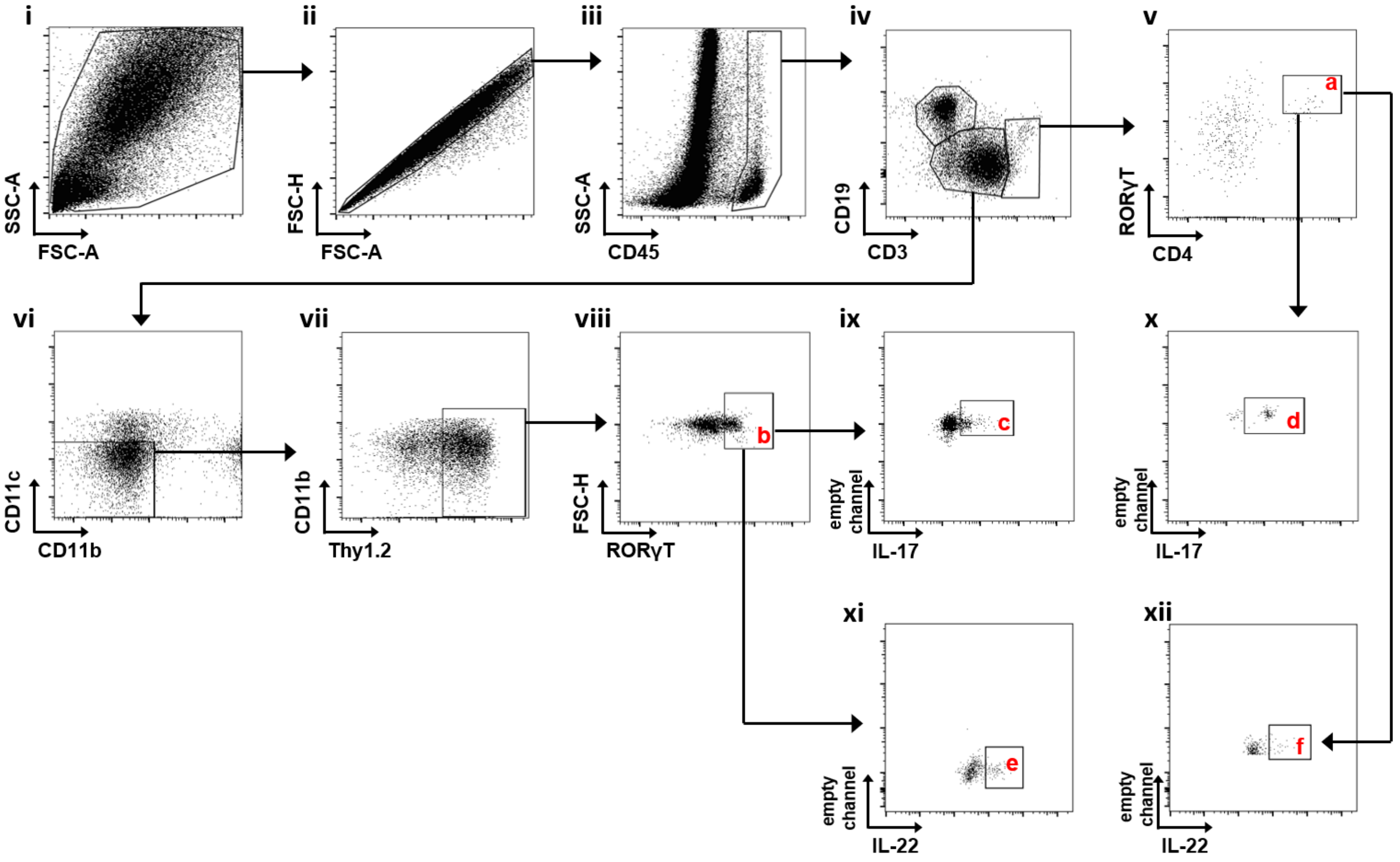

Supplemental Figure 1. Representative plots showing gating strategy used for the identification of Th17 cells and ILC3 by flow cytometry. Cells were selected by forward scatter (FSC) and side scatter (SSC) profile (i) and excluding doublets (ii). After gating hematopoietic cells by CD45 expression (iii), cells were distinguished by the expression of CD3 (iv). Th17 cells were identified as CD3+CD4+RORγt+ cells (v; a). ILC3 were identified as CD45+CD3-CD19-CD11b-CD11c-Thy1.2+RORγt+ (viii; b). IL-17-producing cells within ILC3 and Th17 were identified in (ix; c) and (x; d), respectively. IL-22-producing cells within ILC3 and Th17 were identified in (xi; e) and (xii; f), respectively.

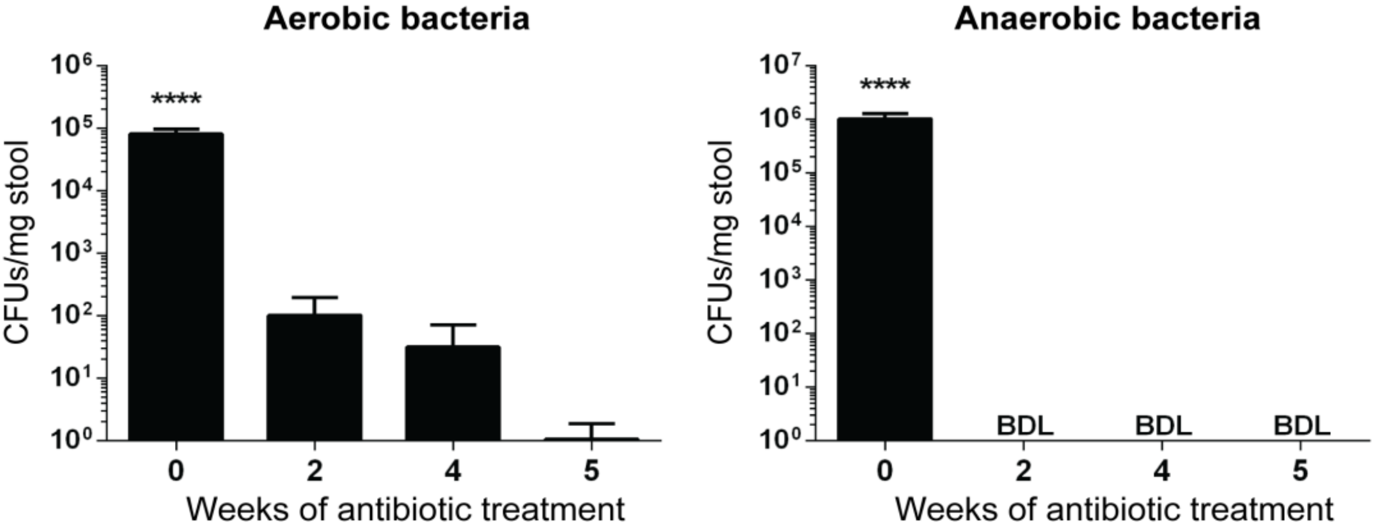

Supplemental Figure 2. Rate of microbiota depletion during antibiotic treatment, assessed by quantification of aerobic and anaerobic bacteria colony-forming units (CFU) in stool.

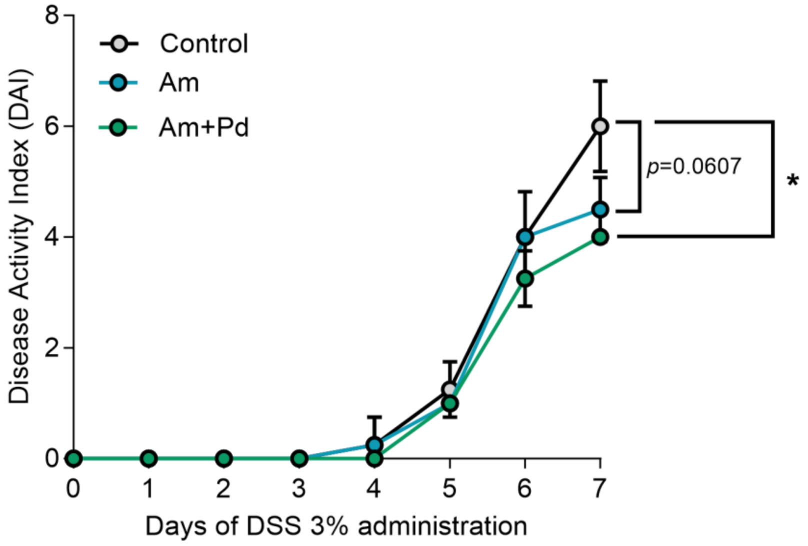

Supplemental Figure 3. Mice were supplemented with *Akkermansia muciniphila* (Am) from murine origin (isolated from mouse), alone or in combination with *Parabacteroides distasonis* (Am+Pd), during 12 days by daily oral gavage, followed by administration of 3% DSS for 7 days. Disease progression was assessed by scoring the disease activity index (DAI) throughout the experiment. Data is presented as mean ± standard deviation (SD). Statistically significant values are: $*p < 0.05$.

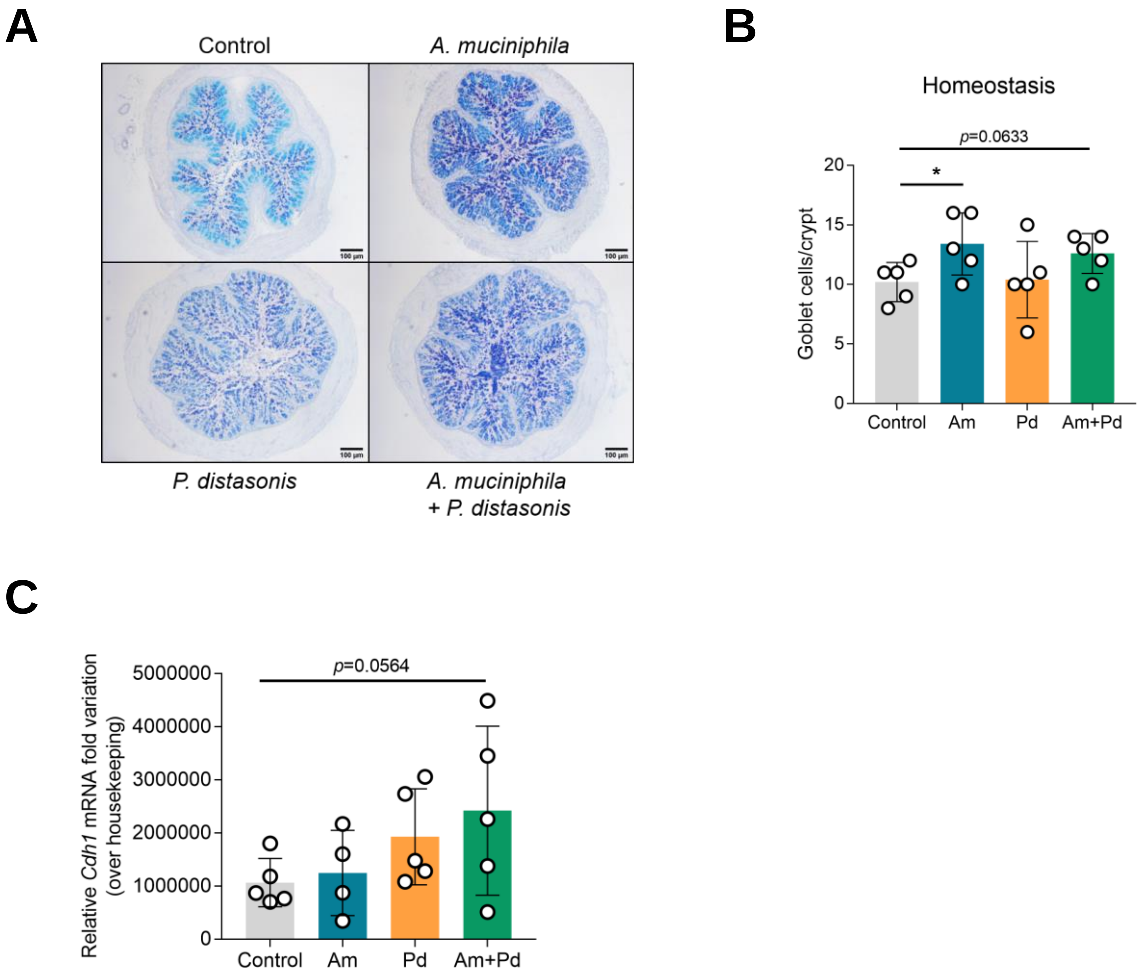

Supplemental Figure 4. (A-B) Quantification of goblet cell numbers per crypt in mice supplemented and non-supplemented, at homeostasis. **(C)** Expression of *Cdh1* in colonic tissue from mice supplemented and non-supplemented, at homeostasis, was analysed by qPCR. N=5 per group. Data is presented as mean ± standard deviation (SD). Statistically significant values are: *p < 0.05; **p < 0.01.

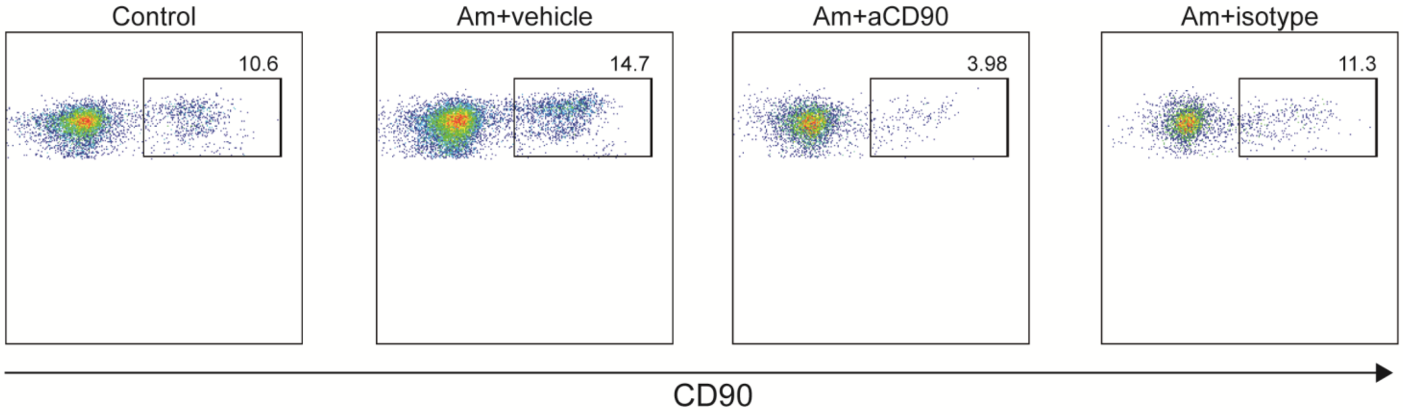

Supplemental Figure 5. Anti-CD90 treatment reduces the number of ILCs in the colon. Flow cytometry analysis of lamina propria leukocytes isolated from mice supplemented with *Akkermansia muciniphila* (Am), and treated with anti-CD90 monoclonal antibody (Am+aCD90), an isotype control antibody (Am+isotype), or no antibody treatment (Am+vehicle). Control group is related to mice that were not supplemented or treated. Representative dot-plots are shown.
