# Supplementary material for: Akkermansia muciniphila and Parabacteroides distasonis synergistically protect from colitis by promoting ILC3 in the gut
Source: mBio. 2024 Mar 12;15(4):e00078-24. doi: 10.1128/mbio.00078-24 (PMC11210198; doi:10.1128/mbio.00078-24)
Supplement: Supplemental Tables — Tables S1 and S2. [file mbio.00078-24-s0002.docx]

| **Feature** | **ANCOMBC.**  **pval** | **ANCOMBC.**  **adjpval.fdr** | **mean.**  **Resistant** | **mean.**  **Susceptible** | **log2FC** |
| --- | --- | --- | --- | --- | --- |
| *Odoribacter* | 8.46E-185 | 5.92E-183 | 0 | 3.69260087 | -11.796541 |
| *Ureaplasma* | 2.50E-157 | 5.83E-156 | 0.64682816 | 0 | 9.28526621 |
| *Flavonifractor* | 3.47E-77 | 6.07E-76 | 0.00122158 | 0.88247151 | -8.6108253 |
| *Clostridium_IV* | 2.49E-45 | 3.48E-44 | 0 | 2.48836442 | -11.227297 |
| *Helicobacter* | 8.81E-44 | 1.03E-42 | 1.40482037 | 0.0014659 | 9.13286018 |
| *Prevotella* | 3.72E-29 | 3.26E-28 | 0 | 4.23205189 | -11.993209 |
| *Pseudoflavonifractor* | 1.14E-28 | 8.85E-28 | 0.00061079 | 1.2079012 | -9.5178152 |
| *Lactococcus* | 6.09E-24 | 4.26E-23 | 0.02259928 | 0 | 4.50872692 |
| *Clostridium_XI* | 2.82E-23 | 1.79E-22 | 0.11116405 | 0 | 6.75567307 |
| *Escherichia_Shigella* | 1.98E-19 | 9.88E-19 | 4.96512381 | 0.00659655 | 9.34530766 |
| *Bacteroides* | 2.46E-19 | 1.15E-18 | 21.4167919 | 2.19884927 | 3.28331061 |
| *Alloprevotella* | 7.85E-18 | 3.23E-17 | 0 | 0.45442885 | -8.7769168 |
| *Clostridium_XlVb* | 2.72E-17 | 1.06E-16 | 0 | 0.09528347 | -6.5355041 |
| *Anaeroplasma* | 4.56E-17 | 1.68E-16 | 0 | 1.73782387 | -10.709641 |
| *Clostridium_XlVa* | 4.37E-13 | 1.46E-12 | 39.2152551 | 9.47264265 | 2.04945619 |
| *Parabacteroides* | 6.11E-13 | 1.94E-12 | 5.65715054 | 0.14072635 | 5.31877011 |
| *Oscillibacter* | 7.56E-13 | 2.30E-12 | 0.00061079 | 1.02686261 | -9.2837743 |
| *Parasutterella* | 1.27E-08 | 3.55E-08 | 1.57828514 | 0.33422509 | 2.23593966 |
| *Akkermansia* | 3.01E-08 | 8.11E-08 | 12.0661854 | 1.29292337 | 3.22122696 |
| *Desulfovibrio* | 1.27E-07 | 3.18E-07 | 0.47030943 | 0.08282332 | 2.49070848 |
| *Insolitispirillum* | 1.48E-07 | 3.56E-07 | 0.00671871 | 0 | 2.90122185 |
| *Desulfomicrobium* | 9.03E-06 | 1.91E-05 | 0.25714321 | 0 | 7.95795556 |
| *Holdemania* | 0.000225865 | 0.00045173 | 0 | 0.25213472 | -7.9296935 |
| *Vampirovibrio* | 0.000288407 | 0.000560791 | 0 | 0.03811339 | -5.2367177 |
| *Alistipes* | 0.000447334 | 0.000846308 | 0.95100231 | 12.5678895 | -3.7226946 |
| *Tannerella* | 0.000937811 | 0.001683251 | 0.0140482 | 0.0058636 | 1.12818827 |

**Supplemental Table 1.** **Most significant hits found in the 16S rRNA sequencing using the ANCOM-BC test for susceptible and resistant mice groups.**

**Supplemental Table 2.** **Most significant hits found in the 16S rRNA sequencing using the ANCOM-BC test for susceptible and susceptible+FMT mice groups.**

| **Feature** | **ANCOMBC.**  **pval** | **ANCOMBC.**  **adjpval.fdr** | **mean.**  **Susceptible** | **Mean.**  **Susceptible+FMT** | **log2FC** |
| --- | --- | --- | --- | --- | --- |
| *Helicobacter* | 1.37E-59 | 9.87E-58 | 0.0014659 | 3.07838898 | -10.492168 |
| *Clostridium_XI* | 1.30E-36 | 3.11E-35 | 0 | 0.0714626 | -6.7463616 |
| *Alloprevotella* | 9.61E-33 | 1.73E-31 | 0.454428849 | 0 | 9.40378673 |
| *Clostridium_XVIII* | 3.19E-10 | 3.83E-09 | 0.002198849 | 0.0641331 | -4.4966201 |
| *Anaerostipes* | 7.90E-10 | 8.12E-09 | 0.128999157 | 0.00427554 | 4.71203865 |
| *Holdemania* | 2.39E-08 | 2.15E-07 | 0.252134716 | 0 | 8.55563492 |
| *Ureaplasma* | 1.66E-07 | 1.33E-06 | 0 | 0.02748562 | -5.3891913 |
| *Coprococcus* | 2.25E-07 | 1.62E-06 | 0 | 0.01099425 | -4.1179972 |
| *Eubacterium* | 1.48E-06 | 9.70E-06 | 0.063033679 | 0.00061079 | 5.63420602 |
| *Akkermansia* | 3.00E-06 | 1.80E-05 | 1.29292337 | 15.5489183 | -3.5874184 |
| *Clostridium_IV* | 5.95E-06 | 3.29E-05 | 2.488364423 | 0.34265401 | 2.85793674 |
| *Olsenella* | 9.53E-05 | 0.000489875 | 0.00073295 | 0.01038346 | -2.9762839 |
| *Clostridium_XlVb* | 0.000115277 | 0.000553331 | 0.095283468 | 0.28523961 | -1.5751336 |
| *Turicibacter* | 0.000123892 | 0.000557514 | 0.064499579 | 0.88320446 | -3.7615325 |
| *Alistipes* | 0.000215537 | 0.000823978 | 12.56788947 | 4.36715897 | 1.52483063 |
| *Dorea* | 0.000217439 | 0.000823978 | 0.016124895 | 0.00183237 | 2.7457358 |
| *Tannerella* | 0.000405591 | 0.001460128 | 0.005863598 | 0.04764173 | -2.8860669 |
| *Parabacteroides* | 0.001121515 | 0.003608068 | 0.140726353 | 0.78059149 | -2.466045 |
| *Flavonifractor* | 0.001152577 | 0.003608068 | 0.882471507 | 0.41289503 | 1.09452695 |
